# HIV seroconversion among female sex workers: retrospective cohort study from a large-scale HIV prevention and sexual and reproductive health program in Tanzania

**DOI:** 10.3389/frph.2024.1332236

**Published:** 2024-06-11

**Authors:** Gaspar Mbita, Amasha Mwanamsangu, Albert N. Komba, Caterina Casalini, Maneno Luponya, Kelly Curran, Alice Christensen, Young-Mi Kim, Jason Reed, Neema Makyao, Upendo Kategile, Donaldson F. Conserve, Jos van Roosmalen, Thomas van den Akker

**Affiliations:** ^1^ Jhpiego, Dar-es-Salaam, Tanzania; ^2^ Athena Institute, Vrije Universiteit, Amsterdam, Netherlands; ^3^ Jhpiego, Monrovia, Liberia; ^4^ Jhpiego, Baltimore, MD, United States; ^5^ Ministry of Health Community Development, Gender, Elderly, and Children, National AIDS Control Program, Dodoma, Tanzania; ^6^ USAID, Dar es Salaam, Tanzania; ^7^ Department of Prevention and Community Health, Milken Institute School of Public Health, George Washington University, Washington, DC, United States; ^8^ Department of Obstetrics and Gynecology, Leiden University Medical Center, Leiden, Netherlands

**Keywords:** Tanzania, female sex workers (FSWs), key populations, HIV seroconversion, HIV/AIDS (acquired immunodeficiency syndrome)

## Abstract

**Introduction:**

In 2016, UNAIDS set ambitious targets to reduce global HIV infections by 75% by 2020 and 90% by 2030, based on the 2.1 million new infections reported in 2010. However, by 2022, new HIV infections had only decreased by 38%, from 2.1 million in 2010 to 1.3 million in 2022, raising concerns about reaching the 2030 goal. Female sex workers (FSWs) in sub-Saharan Africa face a disproportionately high risk of HIV acquisition, contributing 5%–20% of all new infections in several countries in the region. This analysis investigates HIV seroconversion and associated factors among FSWs, offering insights into critical interventions for preventing HIV transmission in this population and advancing the goal of ending the HIV pandemic by 2030.

**Methods:**

We conducted a retrospective cohort study involving 17,977 FSWs who initially tested HIV negative upon enrollment in the Sauti project between October 2016 and September 2018. HIV incidence rates were calculated by dividing the number of new HIV cases by observed person-time within the cohort. Cox regression analysis identified factors associated with seroconversion.

**Results:**

The study revealed an HIV incidence rate of 8.6 per 100 person-years among FSWs [95% confidence interval (CI): 8.1–9.1]. Factors independently associated with HIV seroconversion included age 35 years or older [adjusted hazard ratio (aHR): 2.53; 95% CI: 2.03–3.14], unprotected sex (aHR: 1.27; 95% CI: 1.13–1.42), STI symptoms (aHR: 1.99; 95% CI: 1.67–2.38), and alcohol consumption before sex (aHR: 1.20; 95% CI: 1.07–1.34).

**Conclusion:**

Targeted interventions are vital in curbing HIV transmission among FSWs, with a focus on expanding access to primary HIV prevention services, particularly for older FSWs who face heightened risk. Tailored sexual health education programs are imperative to encourage consistent condom use and enable informed decision-making. Accessible and timely STI screening and treatment services are crucial to mitigate HIV transmission risk. Collaborative partnerships between healthcare providers, community organizations, and government agencies are essential in implementing these interventions among FSWs.

## Introduction

The ongoing global HIV pandemic poses a persistent challenge, affecting millions of individuals worldwide. Since the onset of the epidemic, approximately 85.6 million people have been infected with HIV, resulting in about 40.4 million lives lost to HIV-related causes. As of the end of 2022, the global population living with HIV reached 39.0 million (1).

The global response to HIV faces considerable challenges in meeting ambitious targets set by UNAIDS. In 2016, the UN General Assembly established goals to reduce new HIV infections by 75% by 2020, aiming to reduce new infections to fewer than 500,000 and by 90% by 2030, aiming for fewer than 200,000 new infections globally (2, 3). However, by 2022, new infections had only decreased by 38%, from 2.1 million to 1.3 million, falling short of the 2020 target and raising concerns about achieving the 2030 goal (1, 4).

In Tanzania, the annual HIV incidence for adults aged 15 years and older is 0.18%, with females experiencing a higher rate of 0.24% compared to males at 0.11% (5). This translates to an estimated 60,000 new cases of HIV infections occurring each year among both adult females and males aged 15 years and older (5).

One particular population that demands critical attention in the context of HIV preventive efforts is female sex workers (FSWs). The HIV prevalence among FSWs worldwide remains alarmingly high and has seen little change over time, reported at 11.8% in 2014 and slightly declining to 10.4% in 2018 (6). Across sub-Saharan Africa, FSWs face a disproportionately high risk of HIV acquisition, contributing 5%–20% of total new HIV infections in several sub-Saharan countries and potentially impeding progress toward global targets (7).

In Eastern and Southern Africa, FSWs bear a significant HIV burden, with prevalence soaring as high as 33.3% (8). A survey conducted in one region of Tanzania reveals an exceptionally high HIV prevalence of 40.9% among FSWs, which is 8.1 times higher than the prevalence among women aged 15–49 years in the general population (9).

The Tanzania Health Impact Survey is a valuable tool for tracking HIV trends and guiding prevention strategies in the country. However, it lacks specific HIV estimates for key populations, such as FSWs. Other studies assessing HIV incidence and associated factors have been conducted in the study settings, sometimes with smaller sample sizes. Our analysis utilizes a substantial sample size within a routine real-life program delivering community-based HIV combination prevention interventions focusing on FSWs. The findings from this analysis may offer valuable insights into the current body of knowledge, shaping our understanding of the HIV epidemics and contributing to global efforts to end HIV epidemics by 2030.

### Program description

**Figure 1:**
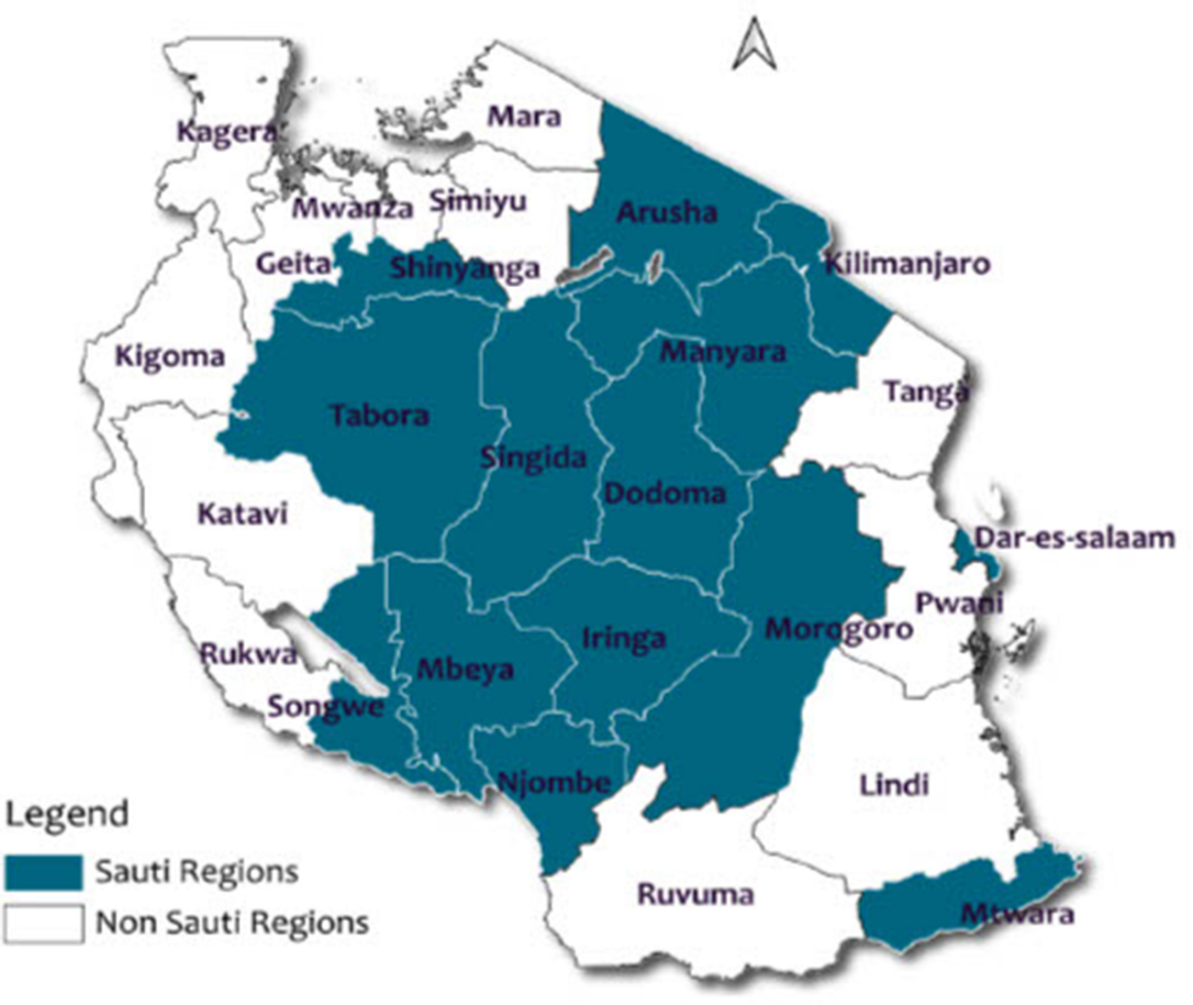
Map of sauti project regions included in the analysis.

This analysis used data from the Sauti Project, a US President's Emergency Plan for AIDS Relief (PEPFAR) funded, United States Agency for International Development (USAID) administered, community-based project that provided comprehensive HIV prevention, treatment, and sexual and reproductive health care, including contraceptive services, to key and vulnerable populations in Tanzania (10–13). Jhpiego, an affiliate of Johns Hopkins University, implemented the Sauti Project with its partners EngenderHealth, Inc., Pact. Inc. and the Tanzania National Institute for Medical Research. Sauti Project provided HIV prevention services in 14 of 26 regions of Tanzania's mainland between October 2015 and January 2020 (Figure 1).

## Materials and methods

### Study design and setting

We utilized a retrospective cohort study design to examine HIV seroconversion and associated factors among FSWs participating in the Sauti project between October 2016 and September 2018. During this period, the Ministry of Health, Community Development, Gender, Elderly, and Children (MoHCDGEC) designated 14 regions on Tanzania's mainland to the Sauti Project to implement community-based interventions, as reported in the previous studies (10, 11). These regions included Dodoma, Singida, Tabora, Dar es Salaam, Morogoro, Mtwara, Shinyanga, Arusha, Kilimanjaro, Manyara, Iringa, Mbeya, Njombe, and Songwe (Figure 1). Based on the 2022 Population and Housing Census conducted in the United Republic of Tanzania, these regions collectively represent 54% of the entire population residing in mainland Tanzania (14).

The Sauti Project served a diverse group, including FSWs, clients of FSWs (CFSWs), men who have sex with men (MSM), vulnerable adolescent girls, and young women (aged 15–24 years, sexually active, and either not attending school or having dropped out for at least a month), children of FSWs (aged 0–14 years), and individuals residing in areas with a high risk of HIV transmission known as hotspots. These hotspots included brothels, mining and fishing villages, plantations, truck drivers’ stops, and social venues like bars, nightclubs, and guesthouses. The project provided comprehensive clinical care, including HIV testing, facilitated linkage to HIV care and treatment, sexual HIV risk assessment, condom distribution, family planning, and screening for sexually transmitted infections (STIs), tuberculosis (TB), substance abuse, and gender-based violence (GBV). Additionally, it offered referrals for post-GBV care, encompassing social, legal, and medical assistance.

### Study population and sampling

The participants in this analysis were FSWs aged 18 years or older who reported exchanging sex for cash or goods as their primary source of income, making up at least half of their monthly earnings (10–12). We used a participant-specific alphanumeric unique identification code (UIC) to link records of subsequent visits of the same client receiving services across project sites.

The Sauti Project employed an approved data collection tool from the MoHCDGEC. De-identified data were regularly entered into a project monitoring database for analysis. A team of health providers trained in ethics, informed consent, and data collection procedures conducted daily HIV testing at the Sauti Project sites. Information was collected during in-person services and recorded using routine project monitoring tools. A dedicated data manager verified the data daily alongside field supervisors to maintain accuracy.

**Figure 2:**
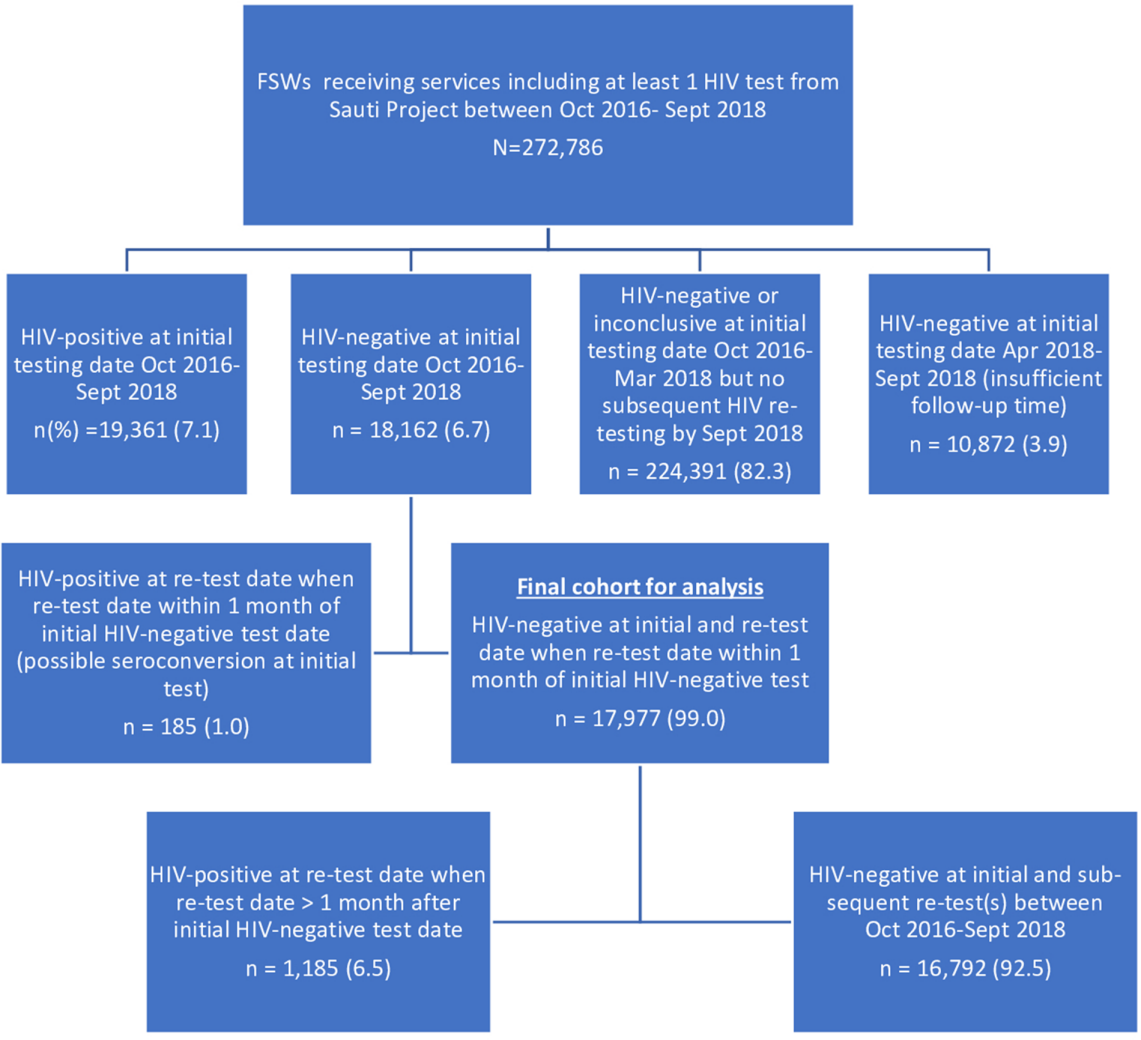
A cohort of FSWs constructed from the sauti project's database, October 2016–September 2018. *We included FSWs who attended both initial and subsequent visits between October 2016 and September 2018 and excluded those who had a second HIV test within four weeks of their initial test to avoid potential inclusion of individuals in the window period.

We established a cohort of 18,162 FSWs who received an initial HIV-negative test result and received at least one follow-up HIV test at a subsequent visit to the Sauti Project. We excluded 185 FSWs who had a positive follow-up HIV test within four weeks of their initial HIV-negative test to avoid the potential inclusion of those who may have been seroconverting at the time of their initial negative HIV test. Finally, all participants in the cohort had at least six months of possible follow-up time. Therefore, we excluded those who had their first negative HIV test in or after April 2018, which was six months before September 2018 (the end of the analysis period). Our final analysis sample consisted of 17,977 FSWs with complete records of HIV testing information throughout the follow-up period (Figure 2).

### Study variables and analysis

We measured various sociodemographic and behavioral variables, including age, highest education level attained, condom use in the last three sexual acts, marital status, history of anal sex, syndromic STI screening results, and alcohol consumption before sexual activity.

The main outcome of interest was HIV seroconversion, defined as a change in HIV status from negative to positive during retesting for HIV at least four weeks after an initial negative test result within the analysis period (15).

We performed statistical analyses using STATA version 15 (StataCorp, College Station, TX, USA). We used medians and interquartile ranges (IQRs) for continuous variables and proportions for categorical variables to describe baseline sociodemographic and behavioral characteristics. Proportions of HIV seroconversion after the first HIV testing were estimated using Kaplan-Meier methods. Cox proportional hazards regression models were used to determine HIV seroconversion and associated risk factors. We included all the variables associated with HIV seroconversion in the bivariate analysis (at *p* < 0.20 level) in the multivariable Cox regression model. Associations were estimated using hazard ratios with 95% confidence intervals (CI). Finally, associations were examined at a significance level of *p* < 0.05 (two-sided test). The proportional hazards assumption was checked using graphical and formal methods and Schoenfeld residual tests. The results of these analyses suggested that the proportional hazards assumption was satisfied. We used the Akaike and Bayesian information criteria (AIC/BIC) to assess model parsimony.

### Laboratory tests and methods

We established HIV status using rapid diagnostic tests (RDTs) from the MoHCDGEC. The trained healthcare workers and laboratory professionals used the nationally approved HIV testing algorithm involving HIV rapid diagnostic tests: SD Bioline HIV ½ (Standard Diagnostics Inc., Suwon, Korea) for screening and Uni-Gold Recombigen HIV test (Trinity Biotech, Wicklow, Ireland) for confirmation of infections. We used the Enzyme-linked immunosorbent assay (ELISA) tests, Murex HIV Ag/Ab Combination (DiaSorin S.p.A., UK Branch), and Enzygnost HIV Integral 4 ELISA (Siemens Healthcare, Germany) to resolve discordant results between the two HIV rapid tests (11, 16).

## Ethical oversight

Approval to conduct a secondary analysis of Sauti project data was obtained from the institutional review boards of the Tanzania National Institute of Medical Research (NIMR/HQ/R.8c/Vol.1/678) and Johns Hopkins Bloomberg School of Public Health (IRB No 00006673). A detailed consent script was discussed with each FSW, and each FSW gave written consent for receipt of care, including HIV testing, follow-up contact for care, or contacting partners as needed and using the FSW's de-identified data for project improvement.

## Results

### Sociodemographic characteristics of the FSWs

Data from a cohort of 17,977 FSWs were investigated. In terms of demographics, 12,260 (68.2%) FSWs were aged 25–34 years, 8,212 (45.7%) had completed primary education, 16,626 (92.5%) had tested negative for syndromic STIs, 11,340 (63.1%) reported using a condom sometimes or always in the last three sexual acts, 7,394 (41.1%) FSWs reported ever having had anal sex, and 7,356 (40.9%) reported using alcohol when having sex in the last month (Table 1).

**Table 1:** Sociodemographic and risk behavior characteristics of FSWs receiving repeating HIV testing through sauti project services (*N* = 17,977).

Risk behaviors	Total *n* (%)	HIV seroconversion status *n* (%)	
		Convertors	Non-convertors
Overall	17,977	1,185 (6.6)	16,792 (93.4)
Age (years)			
18–24	2,864 (15.9)	112 (3.9)	2,853 (96.1)
25–34	12,260 (68.2)	785 (6.4)	11,475 (93.6)
35+	2,853 (15.9)	288 (10.1)	2,565 (89.9)
Highest educational level			
Never/some primary	2,251 (12.5)	115 (6.9)	2,136 (93.1)
Primary education	8,212 (45.7)	519 (6.3)	7,693 (93.7)
Secondary/higher	7,514 (41.8)	511 (6.8)	7,003 (93.2)
Condom use in the last three sexual acts			
Sometimes/always	11,340 (63.1)	723 (6.4)	10,617 (93.6)
Never	6,637 (36.9)	462 (7.0)	6,175 (93.0)
Ever had anal sex			
No	10,583 (58.9)	588 (5.6)	9,995 (94.4)
Yes	7,394 (41.1)	597 (8.1)	6,797 (91.9)
Syndromic STI screening result			
Negative	16,626 (92.5)	1,049 (6.3)	15,577 (93.7)
Positive	1,351 (7.5)	136 (10.1)	1,215 (89.9)
Alcohol use when had sex			
No	10,621 (59.1)	602 (5.7)	10,019 (94.3)
Yes	7,356 (40.9)	583 (7.9)	6,773 (92.1)

### HIV seroconversion rate

Among this cohort of 17,977 FSWs, 38,037 additional tests were conducted in the follow-up period, or an average of 2.1 tests per FSW. We observed a total of 1,185 (6.6%) seroconversions occurring >1 month after the baseline HIV-negative test result. The total follow-up duration was 13,822.9 person-years (PY) at risk. The median follow-up time was 7.8 months, with an interquartile range of 4.4 to 13.4 months. Overall, the seroconversion rate was 8.6 per 100 PY.

**Figure 3:**
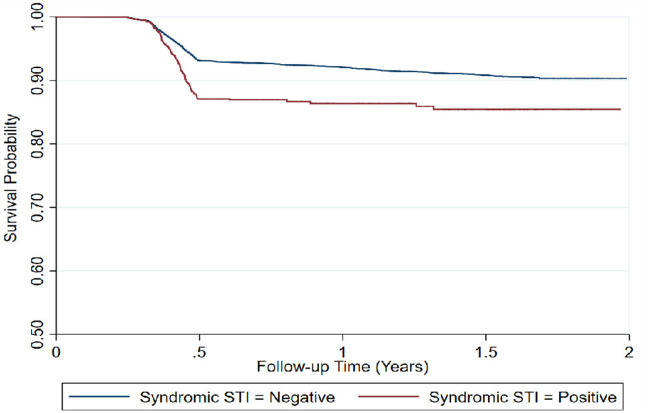
Time to HIV infection among repeat testing FSWs (*n* = 17,997), by syndromic STI screening results.

Higher rates of seroconversion were observed among FSWs aged 35 years and above (20.6/100 PY at risk), those syndromically diagnosed with STI (16.1/100 PY at risk), as well as those who reported never using condoms in the last three occasions of sexual intercourse (9.1/100 PY at risk) (Table 2) and (Figure 3).

**Table 2:** HIV incidence estimates among FSWs receiving repeating HIV testing through sauti project services by sociodemographic and risk behavior characteristics (*N* = 17,977).

Sociodemographic and risk behaviors	HIV seroconversions (D)	Person years at risk of follow-up (Y)	Incidence rate per 100 PY at risk (R)	95% CI
Overall	1,185	138.2	8.6	8.1–9.1
Age (years)				
18–24	112	13.7	8.2	6.8–9.8
25–34	785	110.5	7.1	6.6–7.6
35+	288	14.0	20.6	18.4–23.1
Highest educational level				
Never/some primary	115	12.6	12.3	10.5–14.4
Primary education	519	42.9	12.1	11.1–13.2
Secondary/higher	511	82.6	6.2	5.7–6.7
Condom use in the last three sexual acts				
Sometimes/always	723	91.9	7.9	7.3–8.5
Never	462	46.4	9.1	9.1–10.9
Ever had anal sex				
No	588	65.9	8.2	7.6–8.9
Yes	597	72.3	8.9	8.2–9.7
Syndromic STI screening result				
Negative	1,049	129.8	8.1	7.6–8.6
Positive	136	8.4	16.1	13.6–19.1
Alcohol use when having sex				
No	602	76.5	7.7	7.3–8.5
Yes	583	61.7	9.4	8.7–10.2

D: number of participants who seroconvert, Y: person-years of follow-up, R: rate per person-years of follow-up.

### Risk factors for HIV seroconversion

In the multivariable regression, age, education, condom use, symptoms of STI, and having used alcohol during sex were independent risk factors for HIV seroconversion. The strongest HIV risk factor was being older: FSWs aged 35 years or more had 2.5 times higher risks compared to those aged 18–24 years [aHR 2.53; 95% CI 2.03–3.14]. FSWs who reported not using a condom in the last three sexual acts had a 1.3 times higher risk compared to those who sometimes/always used a condom [aHR 1.27; 95% CI 1.13–1.42]. Those who attained secondary education or higher had the lowest risks (aHR 0.51; 95% CI 0.42–0.63) compared to those who did not complete any schooling or some primary education. Having syndromic STI symptoms doubled the HIV risk compared to those without STI symptoms [aHR 1.99; 95% CI 1.67–2.38], and alcohol use during sex increased the risk by 1.2 [aHR 1.20; 95% CI 1.07–1.34] (Table 3).

**Table 3:** Factors associated with HIV seroconversion among FSWs receiving repeat HIV testing through sauti project services by multivariable proportional hazard regression (*N* = 17,977).

Sociodemographic and risk behaviors	HIV incidence rate per 100 PY at risk	Crude analysis		Adjusted analysis	
		cHR [95% CI]	*p*-value	aHR [95% CI]	*p*-value
Age (years)					
18–24	8.2	Reference		Reference	
25–34	7.1	0.87 [0.71–1.06]	0.169	1.07 [0.87–1.32]	0.530
35+	20.6	2.53 [2.03–3.14]	<0.001	2.44 [1.96–3.04]	<0.001
Highest educational level					
Never/some primary	12.3	Reference		Reference	
Primary education	12.1	0.98 [0.82–1.18]	0.862	0.94 [0.79–1.13]	0.538
Secondary/higher	6.2	0.50 [0.42–0.60]	<0.001	0.51 [0.42–0.63]	<0.001
Condom use in the last three sexual acts					
Sometimes/always	7.9	Reference		Reference	
Never	9.1	1.27 [1.13–1.42]	<0.001	1.26 [1.06–2.27]	0.047
Ever had anal sex					
No	8.2	Reference		Reference	
Yes	8.9	1.08 [0.96–1.21]	0.187	1.20 [1.05–1.36]	0.006
Syndromic STI screening results					
Negative	8.1	Reference		Reference	
Positive	16.1	1.99 [1.67–2.38]	<0.001	1.82 [1.52–2.19]	<0.001
Alcohol use before sex					
No	7.7	Reference		Reference	
Yes	9.4	1.20 [1.07–1.34]	0.002	1.37 [1.22–1.55]	<0.001

HR, hazard ratio.

## Discussion

We applied a unique way of measuring HIV seroconversion using data drawn from a routine, community-based HIV prevention and sexual and reproductive health project, revealing a high HIV seroconversion rate of 8.6 per 100 person-years among FSWs. Factors associated with HIV seroconversion included being aged 35 years or older, engaging in unprotected sex, experiencing STI symptoms, and consuming alcohol before sex. FSWs who had completed secondary school or higher exhibited a lower risk of HIV seroconversion compared to those who never attended school or completed primary education. These findings underscore the importance of tailored interventions for FSWs to prevent HIV acquisition and transmission to their sexual partners.

The rate of HIV seroconversion in this analysis is closer to the incidence (10.4%) reported in a previous community randomized trial in two communities in Iringa among FSWs (17). However, the seroconversion reported in our analysis is higher than the 3.45 per 100 person-years reported in Dar es Salaam in 2022 (18). The disparity in methodological sampling may explain differences in HIV seroconversion in these studies. The previous studies employed cross-sectional surveys, each with a smaller sample size, conducted exclusively in one region of Tanzania (17, 18).

Globally, using alcohol is common among FSWs and is associated with HIV acquisition (19, 20). Nearly half of the FSWs in our analysis consumed alcohol before engaging in a sex act, and HIV seroconversion among such participants was higher than among those who did not use alcohol. A causal link between alcohol and sexual risk behaviors was observed in a previous study (21). The practice of high-risk social behavior, such as alcohol use, provokes physiological and emotional alterations, which are correlated with a low perceived risk of HIV infection and increasing exposure to risks and unprotected sex acts (19–23). Alcohol may have negatively impacted the motivation of FSWs to insist on condom use by having weaker sexual communication and negotiation skills or incorrect use of condoms, resulting in an increased risk of HIV infection. Integrating strategies discouraging same-day alcohol and sexual activity in HIV programming, along with promoting proper PrEP usage, can play a crucial role in reducing HIV infection rates among FSWs and in the general population as well (23, 24).

The prevalence of self-reported STI symptoms among FSWs was 7.5%, similar to the findings of another study (25). The most common STI symptoms were vaginal discharge (curd-like and non-curd-like), vulvar itching, burning, and micturition, being the most significant determinants of HIV infection (26). These findings suggest an association between behaviors resulting in both HIV and STIs, including a possible role of STIs as potentiators of HIV transmission (18, 25, 27). Consequently, we recommend that HIV testing programs reaching FSWs integrate STI assessment and treatment alongside HIV prevention and treatment options.

Although two-thirds of participants were under 35, the observed HIV seroconversion rate was highest among those aged 35 and above, standing at 20.0 per 100 person-years, aligning with findings from other studies (28–30). This phenomenon may be attributed to their prolonged engagement in sex work activities, exposing them to cumulative risks that involve both engaging in unprotected sex and an increased likelihood of encountering potential clients who are HIV-positive, ideas supported by previous studies (31, 32). Other contributors could include a diminished ability of older sex workers to negotiate condom use with clients, heightening their vulnerability to HIV. Older FSWs might encounter more challenges in enforcing condom use, particularly when compared to their younger counterparts (33–35).

Efforts to curtail HIV acquisition among FSWs demand a strategic pivot towards prioritizing men as a focal point in prevention and treatment initiatives (36, 37). The dynamics of HIV transmission within the context of FSWs underscore the critical role that men play in shaping the trajectory of this epidemic. In many settings, men are often less inclined to seek healthcare compared to women, thereby contributing to potential risks for women's health (38). This behavior is particularly relevant in the context of HIV transmission, as the virus is more readily transmitted from men to women than in the reverse direction (39).

## Strengths and limitations

Our study possesses several strengths, particularly in its innovative design and the richness of its sample size. One of the key strengths lies in utilizing real-life program data from a large-scale, community-based HIV and sexual and reproductive health program in Tanzania. This approach offers a unique way of looking at HIV seroconversion among FSWs, allowing for insights derived from the day-to-day implementation of comprehensive HIV prevention and care services. By leveraging data from such a program, our study captures the complexities and nuances of FSWs’ lived experiences, providing a more holistic understanding of the factors influencing HIV transmission dynamics in this population. Additionally, the substantial sample size enhances the robustness of our analysis, ensuring sufficient statistical power to detect meaningful associations and trends.

One limitation of our study is its reliance on retrospective data from a real-life program that employed a standardized national data collection tool. This methodology could potentially introduce unmeasured confounding factors, influencing the observed associations. Additionally, while efforts were made to ensure the representativeness of the study population, it is important to acknowledge that our findings may not be universally applicable to all FSWs in Tanzania or other contexts. Furthermore, the study period may not capture long-term trends or variations in HIV seroconversion rates among FSWs beyond the analyzed timeframe.

## Conclusion

The findings of this study underscore the critical need for targeted interventions to reduce HIV transmission among FSWs. Efforts should prioritize expanding access to primary HIV prevention services, with a particular focus on older FSWs who are at increased risk. Comprehensive sexual health education initiatives, tailored to the specific needs of FSWs and delivered in culturally sensitive settings, should be implemented to promote consistent condom use and empower FSWs to make informed decisions about their sexual health. Accessible and timely STI screening and treatment services are also essential, particularly for FSWs experiencing syndromic STI symptoms, to reduce the risk of HIV transmission. Collaborative partnerships between healthcare providers, community organizations, and government agencies are crucial in implementing these interventions, which also include community and structural interventions to address stigma, criminalization, gender-based violence, and financial insecurity.

## Data Availability

The raw data supporting the conclusions of this article will be made available by the authors, without undue reservation.
